# Lansoprazole is an antituberculous prodrug targeting cytochrome *bc*_1_

**DOI:** 10.1038/ncomms8659

**Published:** 2015-07-09

**Authors:** Jan Rybniker, Anthony Vocat, Claudia Sala, Philippe Busso, Florence Pojer, Andrej Benjak, Stewart T. Cole

**Affiliations:** 1 Global Health Institute, Ecole Polytechnique Fédérale de Lausanne (EPFL), CH-1015 Lausanne, Switzerland; 2 1st Department of Internal Medicine, University of Cologne, D-50937 Cologne, Germany

## Abstract

Better antibiotics capable of killing multi-drug-resistant *Mycobacterium tuberculosis* are urgently needed. Despite extensive drug discovery efforts, only a few promising candidates are on the horizon and alternative screening protocols are required. Here, by testing a panel of FDA-approved drugs in a host cell-based assay, we show that the blockbuster drug lansoprazole (Prevacid), a gastric proton-pump inhibitor, has intracellular activity against *M. tuberculosis*. *Ex vivo* pharmacokinetics and target identification studies reveal that lansoprazole kills *M. tuberculosis* by targeting its cytochrome *bc*_1_ complex through intracellular sulfoxide reduction to lansoprazole sulfide. This novel class of cytochrome *bc*_1_ inhibitors is highly active against drug-resistant clinical isolates and spares the human H^+^K^+^-ATPase thus providing excellent opportunities for targeting the major pathogen *M. tuberculosis*. Our finding provides proof of concept for hit expansion by metabolic activation, a powerful tool for antibiotic screens.

## Abstract (web summary)

Tuberculosis control is threatened by the continued emergence of drug-resistant strains. Here, Rybniker *et al.* screen a library of FDA-approved drugs and identify a gastric proton pump inhibitor that also has antituberculosis activity and targets the bacterial cytochrome *bc1* complex.

## Graphical abstract

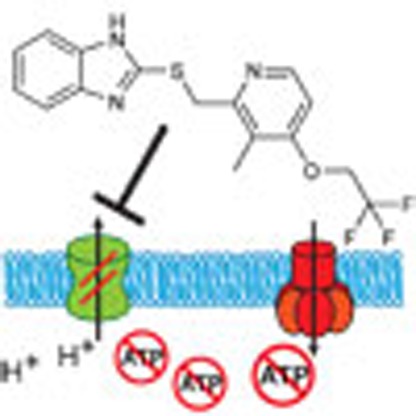

The constant spread of drug-resistant pathogens requires the development of innovative drug-screening methods that generate a substantial amount of lead compounds^1^. The prototype of a difficult-to-treat pathogen with hampered control due to multi-drug resistance (MDR) is *Mycobacterium tuberculosis* (*Mtb*), the causative agent of tuberculosis (TB). Despite greatly increased efforts to identify and develop new therapeutic agents, the TB pandemic continues to be a major cause of morbidity and mortality worldwide. In 2013, TB was responsible for an estimated 1.5 million deaths. In the same year, 480,000 people developed MDR-TB^2^. The outcome data for patients starting MDR-TB treatment in 2011 show a success rate of only 48%, due to lack of effective treatment regimens^2^. To combat this global health problem, the rapid development of safe drugs with new mechanisms of action is required.

In the past decade concerted screening of millions of small molecules for growth inhibition of *Mtb* led to the identification of a few promising compounds, some of which are now in clinical trials^3^. However, due to limitations in both the diversity of chemical libraries and the seemingly limited number of druggable targets, remarkably few truly novel hits were identified in these basic whole-cell screens^4^. Host cell-based phenotypic assays that better reflect the biology of the pathogen may overcome this bottleneck by avoiding the bias of synthetic media-driven target selection^5^,^6^. Another efficient approach to fight notoriously drug-resistant pathogens such as *Mtb* is repurposing of existing drugs, and their analogues, which reduces drug development costs and saves precious time^7^.

When testing Food and Drug Administration (FDA)-approved drugs in an innovative high-throughput screen selecting for compounds that abrogate *Mtb*-induced cytotoxicity^8^, we focused on non-antibiotic hits of clinically approved pharmacophores active against MDR-TB. This screen identified the gastric proton-pump inhibitor (PPI) lansoprazole (LPZ) as a compound with intracellular anti-mycobacterial activity. PPIs, selective inhibitors of the H^+^K^+^-ATPase of the gastric parietal cell, are used extensively for the treatment of acid-related disorders of the stomach^9^. As non-prescription drugs, PPIs have an excellent safety profile and are among the most widely sold drugs in the world^10^.

In this work, we show that LPZ is a prodrug that requires the host's cytoplasm for conversion into a LPZ analogue with antituberculous activity. Successful target identification studies and the fact that the active compound fails to inhibit the human gastric H^+^K^+^-ATPase make this metabolite an attractive lead compound for the TB drug pipeline. Our findings establish host-cell-driven prodrug activation as an additional strategy for successful lead identification, which has a great potential of broadening the spectrum of existing small-molecule libraries.

## Results

### LPZ inhibits growth of intracellular *Mtb*

**Figure 1:**
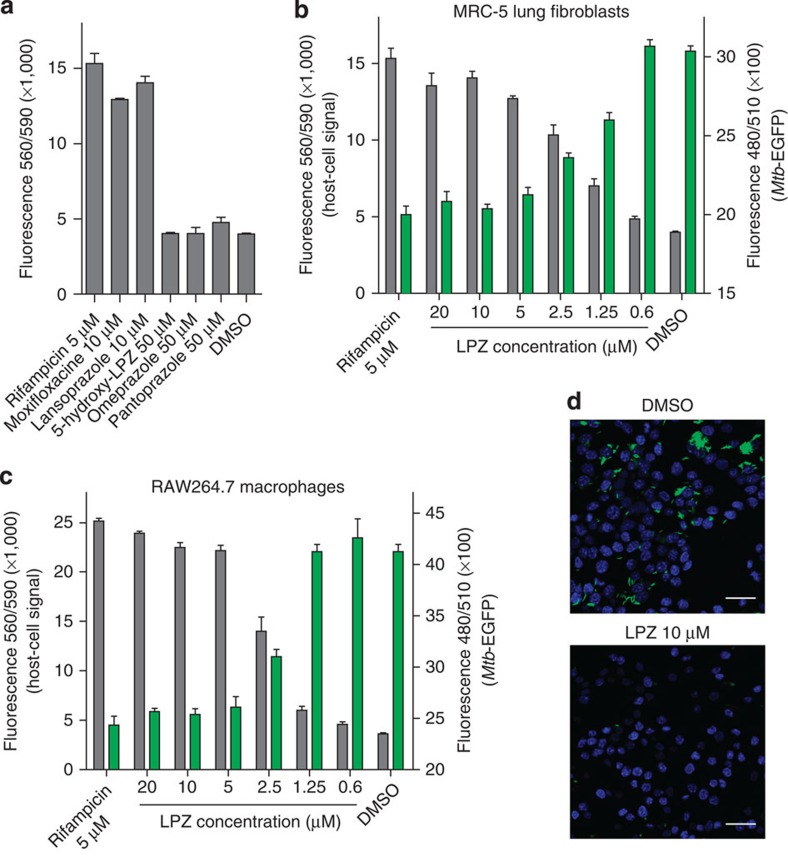
Lansoprazole (LPZ) protects from *Mtb*-induced cytolysis and reduces intracellular bacterial burden. (**a**) Protective activity of LPZ and other drugs against *Mtb*-induced killing of MRC-5 lung fibroblasts. Data are expressed as the mean±s.d. of three individual experiments. Viable fibroblasts were quantified using Prestoblue. (**b**) Dose response of LPZ in the fibroblast survival assay using *Mtb* expressing GFP. Grey bars display host cell survival, green bars quantify intracellular *Mtb*-GFP (mean±s.d. of three independent experiments; right *y* axes are truncated for better visualization). (**c**) Dose response of LPZ in *Mtb*-infected RAW264.7 macrophages. Grey bars display macrophage survival, green bars quantify intracellular *Mtb*-GFP (mean±s.d. of 3 independent experiments; right *y* axes are truncated for better visualization). Growth of intracellular bacteria was inhibited with an IC_50_ of 2.2 μM. (**d**) Confocal microscopy of *Mtb*-GFP-infected RAW264.7 macrophages after treatment with LPZ (10 μM) or vehicle (dimethyl sulfoxide (DMSO)). Macrophage nuclei were stained with 4′,6-diamidino-2-phenylindole (scale bar, 20 μm).

We have recently generated a host cell-based high-throughput screen that selects for compounds that protect MRC-5 lung fibroblasts from *Mtb*-induced cytotoxicity^8^. In this assay, we infect fibroblasts with high multiplicities of infection (MOI of 10) in the presence of screening compounds (Supplementary Fig. 1). After 72 h of co-incubation, the majority of infected fibroblasts are killed by wild-type *Mtb* strains and killing can be quantified by fluorescent staining. Using this assay, we screened 1,280 FDA-approved drugs of the Prestwick chemical library at a concentration of 10 μM thus identifying the gastric PPI LPZ as a potent hit compound that protected fibroblasts at levels comparable to those of well-established anti-mycobacterial drugs (Fig. 1a; Supplementary Table 1).

When confirming LPZ as a hit in the primary screen, we also tested other widely used PPIs such as omeprazole and pantoprazole. Interestingly, these drugs failed to protect fibroblasts at concentrations up to 50 μM (Fig. 1a). This observation made a host-directed immunomodulatory effect leading to better intracellular clearance of *Mtb*, an unlikely mechanism since most PPIs show comparable activity on their eukaryotic target. However, testing 5-hydroxy-LPZ, which was equally inactive at 50 μM (Fig. 1a), suggested that substitutions on the benzimidazole ring may account for the inactivity of these close LPZ analogues (Supplementary Fig. 2).

Hit compound LPZ was tested for growth inhibition of intracellular *Mtb* cells expressing green fluorescent protein (GFP), at different drug concentrations. LPZ reduced the *Mtb*-GFP signal in a dose-dependent manner with a half-maximal inhibitory concentration (IC_50_) of 1.47 μM and this corresponded well with the protection of MRC-5 cells quantified in the same well (Fig. 1b). To rule out a cell-line-specific effect, we performed similar infection experiments using RAW264.7 macrophages in which growth of intracellular bacteria was inhibited with an IC_50_ of 2.2 μM (Fig. 1c,d). In contrast to these results, the anti-mycobacterial activity of LPZ in common mycobacterial broth was 22-fold higher (IC_50_ of 32.8 μM) than its activity in MRC-5 cells (Supplementary Fig. 3).

### LPZ is converted to LPZS intracellularly

**Figure 2:**
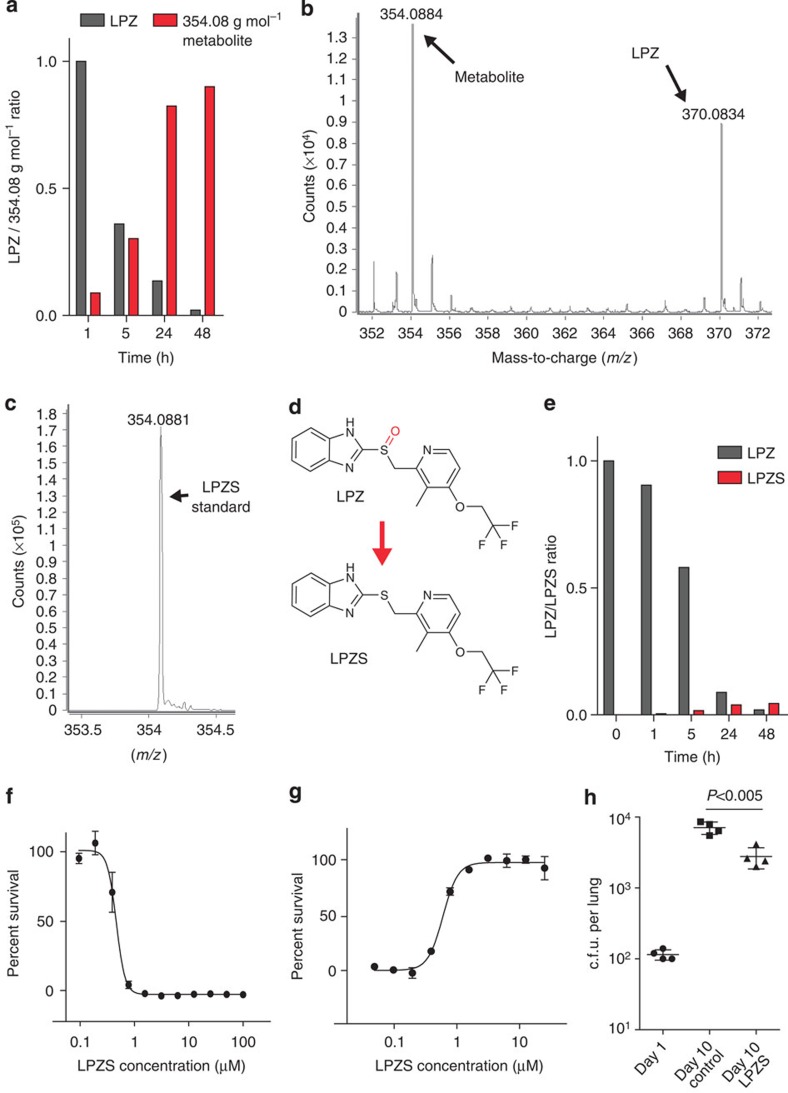
LPZS is a highly selective antituberculous drug with *in vivo* activity. (**a**) Intracellular ratio of LPZ (*m/z* 370.0834, g mol^−1^) and its metabolite (*m/z* 354.0884, g mol^−1^) determined by electrospray ionization quadrupole time-of-flight mass spectrometry (ESI-Q-TOF-MS) over a 48-h period in MRC-5 cells. Representative example of three individual experiments; the complete data set can be found in Supplementary Table 2. (**b**) ESI–MS mass spectra in the range *m/z* 350–375 measured for experiments performed on the cell lysate of MRC-5 fibroblasts exposed to LPZ (extracted ion chromatograms can be found in Supplementary Fig. 4a,b). (**c**) ESI–MS spectrum at *m/z* 354.0884 corresponding to the LPZS standard in methanol. (**d**) Structures of LPZ and LPZS. LPZS is missing the sulfoxide (red), which is essential for LPZ activity on the human proton pump. (**e**) LPZ/LPZS ratio determined by ESI-Q-TOF-MS over a 48-h period in 7H9 broth. Representative example of three individual experiments; the complete data set can be found in Supplementary Table 2. (**f**) Dose–response curve of LPZS for *Mtb* grown in 7H9 broth (mean±s.d. of three individual experiments). (**g**) Survival of *Mtb*-infected MRC-5 fibroblasts was quantified at different concentrations of LPZS (mean±s.d. of three individual experiments). (**h**) Efficacy of LPZS in the mouse model of acute tuberculosis. Bacterial burden (c.f.u.) was determined in the lungs of four mice treated with the vehicle control (TPGS) or four mice treated with LPZS at 300 mg kg^−1^ b.i.d. given by oral gavage (mean±s.d., Student's *t*-test was used to compare groups).

LPZ is a relatively unstable compound that can be modified in both enzymatic and non-enzymatic reactions^11^. Its activity on the human H^+^K^+^-ATPase requires the prodrug LPZ to be converted to a sulfenic acid or sulfenamide intermediate in the acidic environment of the stomach^9^. We reasoned that LPZ may be converted to an active antibiotic primarily in an intracellular environment, thereby explaining the discrepancy between the *ex vivo* and *in vitro* activity. Thus, we quantified intracellular LPZ and possible metabolites over a period of 48 h using liquid chromatography–electrospray ionization/mass spectrometry (LC-ESI/MS) and observed a rapid intracellular decay of LPZ and its near-quantitative conversion to a molecule of lower mass (*m/z* 354.0884, g mol^−1^) (Fig. 2a,b; Supplementary Table 2; Supplementary Fig. 4a). Using analogues as standards, we identified this molecule as lansoprazole sulfide (LPZS), a highly stable LPZ metabolite (Fig. 2c,d; Supplementary Fig. 4b)^11^. LPZS is a precursor for LPZ production that fails to form the sulfenic acid necessary for binding the gastric H^+^K^+^-ATPase^9^,^12^.

Rapid decay of LPZ was also observed in broth; however, LPZS was not the major product (Fig. 2e; Supplementary Table 2). Assuming that LPZS has anti-mycobacterial activity, this differential pattern of LPZ metabolism explains the better activity of the compound during intracellular infection. This hypothesis was confirmed by testing LPZS for growth inhibition of *Mtb* in broth and in intracellular assays. Strikingly, LPZS had a 71-fold improvement of activity compared with LPZ in broth (IC_50_ of 0.46 μM) (Fig. 2f) and showed similar intracellular activity (IC_50_ of 0.59 μM) (Fig. 2g). Thus, intracellular sulfoxide reduction converts LPZ to the potent anti-mycobacterial agent LPZS.

Having established LPZS as a compound with antibacterial activity, we were interested in its antibiotic spectrum. Intriguingly, LPZS showed a highly *Mtb*-selective antibiotic profile with good activity against drug-resistant isolates (Tables 1 and 2). Growth of several Gram-negative and Gram-positive bacteria was not affected by LPZS (Table 1). To determine the physiological significance of these findings, we tested the compound in the murine model of acute TB. Oral administration of LPZS significantly reduced the bacterial burden of *Mtb*-infected mice (Fig. 2h; *in vivo* pharmacokinetic data can be found in Supplementary Fig. 5). There were no signs of toxicity in mice treated with doses as high as 300 mg kg^−1^ b.i.d., owing to the favourable cytotoxicity profile of LPZS (Supplementary Table 3). We also performed *in vitro* drug combination studies with LPZS and several first- and second-line anti-TB drugs, where we observed additive effects for the tested combinations (Supplementary Table 4).

### LPZS targets cytochrome *b*

**Figure 3:**
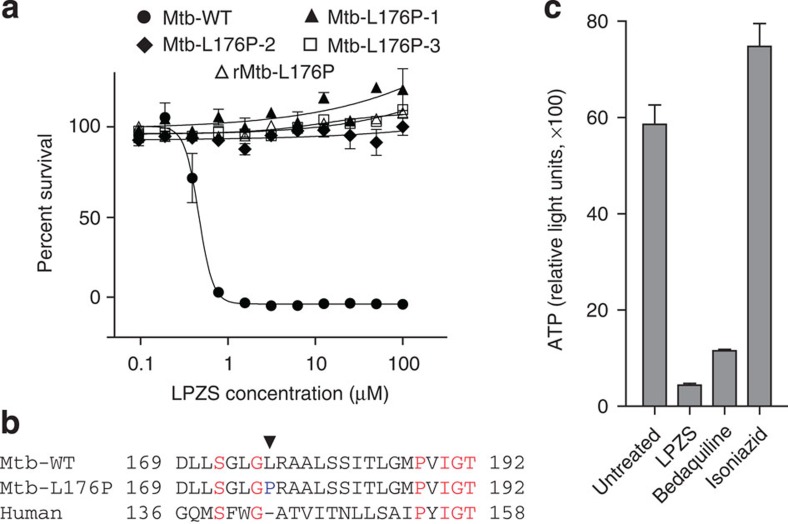
Evidence for LPZS targeting QcrB. (**a**) Dose response of LPZS against wild-type *Mtb*, spontaneous-resistant mutants 1–3 and the genetically engineered recombinant L176P strain (r*Mtb*-L176P) (mean±s.d. of triplicates). (**b**) Mutation in QcrB conferring LPZS resistance. The arrow indicates the L176P mutation that confers resistance to LPZS. The equivalent region of the human QcrB amino-acid sequence is aligned to the *Mtb* sequence. (**c**) LPZS depletes ATP levels after 24 h of treatment (mean±s.d. of three individual experiments). The ATPase inhibitor bedaquiline and the cell-wall inhibitor isoniazid were used as controls. Drug concentrations were 5 × the MIC.

To identify the target of LPZS, we raised drug-resistant mutants of *Mtb* and identified three that displayed stable phenotypic resistance (Fig. 3a). Whole-genome sequencing revealed a single-nucleotide polymorphism (SNP) that changed leucine-176 to proline in the *b*-subunit of the cytochrome *bc*_1_ complex (*qcrB*, Rv2196) in all three mutants (Fig. 3b). Reintroduction of this SNP into wild-type *Mtb* by recombineering confirmed causality (Fig. 3a, r*Mtb*-L176P strain). As an essential respiratory chain component, cytochrome *bc*_1_ (complex III) is required for ATP production. Consistent with QcrB inhibition, we observed massive and rapid ATP depletion in treated *Mtb* (Fig. 3c). ATP levels of the L176P mutant strain were hardly affected by LPZS treatment (Supplementary Fig. 6a). To provide further proof for LPZS targeting cytochrome *bc*_1_, we measured ADP levels in LPZS-treated and untreated bacteria, which allowed us to calculate the ADP/ATP ratio. In untreated wild-type *Mtb*, this ratio was 3.5, LPZS treatment led to a 7-fold increase of intracellular ADP levels upon treatment and an ADP/ATP ratio of 25.8. Most importantly, this effect was not observed when the LPZS-resistant L176P mutant strain was tested (Supplementary Fig. 6b). Thus we provide strong evidence that QcrB is indeed the target protein of the LPZ metabolite LPZS, which is a highly unexpected finding since the gastric H^+^K^+^-ATPase and cytochrome *bc*_1_ are structurally unrelated protein complexes.

### LPZS represents a novel class of QcrB inhibitors

QcrB is an emerging and highly vulnerable drug target of *Mtb*. Recently identified imidazopyridine amides (IPAs) display potent *in vitro* and *in vivo* activity against *Mtb*^13^,^14^. Many data for LPZS are similar to findings obtained with IPA compounds. Both classes share a highly *Mtb*-selective activity profile; they are bacteriostatic in broth and inactive against streptomycin-starved 18b (SS18b), a viable but conditionally non-replicating strain of *Mtb* (Supplementary Fig. 7a,b)^15^. Inactivity against SS18b is most likely due to upregulation of the cytochrome *bd*-oxidase that replaces QcrB, thus avoiding the effect of inhibitors such as LPZS^16^.

**Figure 4:**
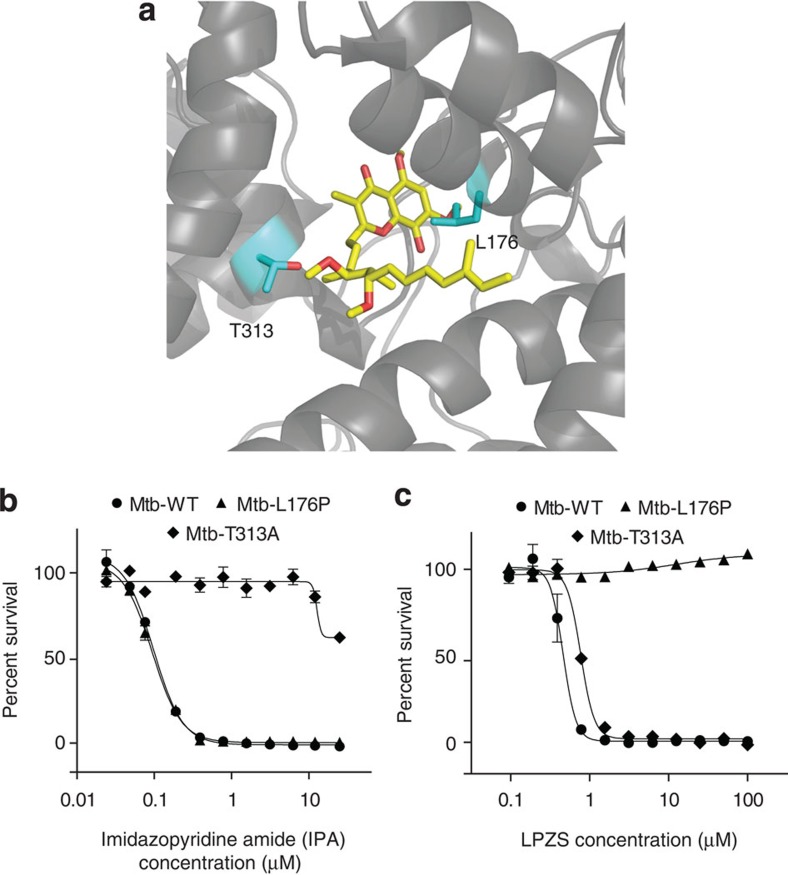
Protein structure model and cross-resistance studies. (**a**) Structure of the *Mtb* QcrB protein homology modelled onto the structure of *R. sphaeroides* QcrB. Close-up of the Q_p_-active site containing the inhibitor stigmatellin A (yellow sticks). Leucine-176, mutated in LPZS-resistant mutants, and threonine 313, mutated in imidazopyridine amide (IPA)-resistant mutants, are depicted as cyan sticks. (**b**) Dose–response curve of the imidazopyridine amide (GSK2111534A) against wild-type Mtb, the L176P mutant and the T313A mutant (mean±s.d. of three individual experiments). (**c**) Dose–response curve showing that the T313A mutant remains susceptible to LPZS (mean±s.d. of three individual experiments).

To visualize both the L176P mutation and the T313A mutation, which confers IPA resistance, we modelled the mycobacterial protein on the published QcrB structure of *Rhodobacter sphaeroides*^17^. Both mutations localized to the ubiquinol oxidation (Q_p_) site close to the Q_p_-inhibitor stigmatellin, which was co-crystalized with the *R*. *sphaeroides* protein (Fig. 4a). This indicates that both compounds target the same QcrB-active site; however, L176P mutants remained susceptible to several IPA compounds (Fig. 4b; Supplementary Table 5). Conversely, growth of the highly IPA-resistant T313A mutant was fully inhibited by LPZS, indicating distinct drug-binding mechanisms for the two drugs (Fig. 4b).

## Discussion

**Figure 5:**
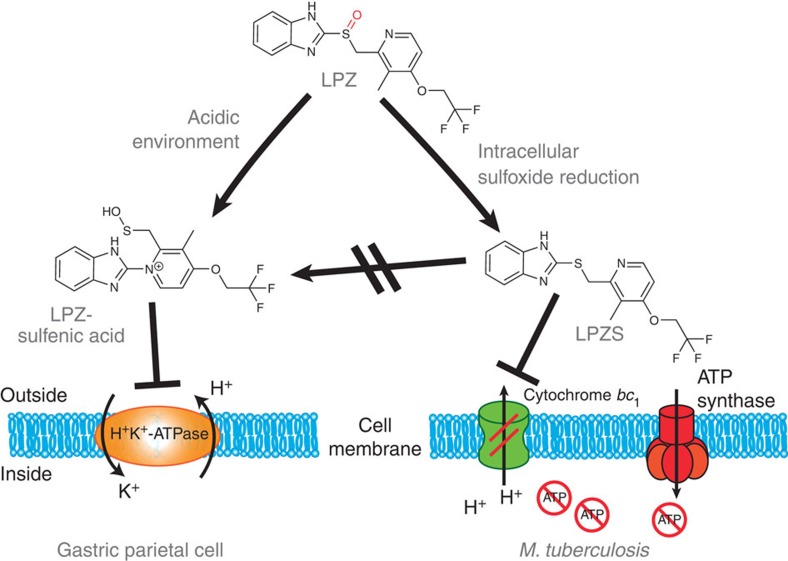
Differential prodrug activation of lansoprazole (LPZ). The proton-pump inhibitor LPZ is converted to a sulfenic acid intermediate in the acidic environment of the gastric gland lumen outside the parietal cell. Further prodrug activation to a sulfenamide (not shown) allows binding to the gastric H^+^K^+^-ATPase and its inhibition. We were able to show that sulfoxide reduction in the cytoplasm of *Mtb*-host cells converts LPZ to the potent antituberculous agent LPZS, which is active against MDR-TB. We provide evidence that LPZS targets cytochrome *bc*_1_ (complex III) leading to disruption of the mycobacterial respiratory chain and rapid ATP depletion. Conversion of LPZS to the sulfenic acid intermediate necessary for inactivation of the gastric H^+^K^+^-ATPase is not possible, making LPZS a highly selective lead compound for the tuberculosis drug pipeline.

In this report, we present the combined use of host cell-based drug screens and *ex vivo* pharmacokinetics, which identified a novel class of antibiotic active against multi-drug-resistant *Mtb*. Our findings have several important implications: first of all and most intriguingly, LPZS is a highly attractive and safe antituberculous lead compound with *in vivo* activity that fails to inhibit the gastric H^+^K^+^-ATPase (Fig. 5). Future structure–activity relationship studies will clearly benefit from 40 years of intense research on PPIs and their analogues. Substantial amounts of *in vitro* and *in vivo* pharmacological data are available for PPI metabolites, thereby providing a rich source for repurposing these substituted benzimidazoles. Analogues with antituberculous activity will have predictable side effects and pharmacokinetic profiles, thereby accelerating translation into pre-clinical and clinical development.

Furthermore, we have confirmed QcrB as a highly specific and druggable *Mtb* target capable of accommodating chemically unrelated compounds with distinct binding mechanisms. The commercially available cytochrome *b* inhibitor LPZS provides a useful tool to further explore this multienzyme complex as a drug target and to study the mycobacterial respiratory chain that is not well understood despite its key role in adaptive processes during intracellular growth.

Several antimalarial drugs such as atovaquone bind the same pocket of plasmodial cytochrome *b*, which indicates that targeting the pathogen's cytochrome *bc*_1_ complex is safe despite the presence of human mitochondrial orthologues^18^. Intriguingly, a recent report identified LPZ-like PPIs as potent inhibitors of plasmodial growth only upon infection of metabolically active liver cells^19^. The authors discuss metabolic transformation of the compounds as a possible cause for their observation, although the underlying mechanism or a drug target was not identified^19^. It is very likely that intracellular sulfoxide reduction, as described in our report, leads to antiplasmodial activity. Thus our findings can improve the future control of both malaria and TB, two leading causes of death.

The prodrug LPZ represents an excellent example of a valuable hit compound in an existing library that was missed by conventional drug screens. Using an innovative screen, we found a new activity for an old drug that supports the notion that novel screening platforms may uncover new antibiotics in old libraries^1^. We provide evidence that intracellular assays are capable of broadening the spectrum of existing small-molecule libraries by identifying drugs that require the host cell environment for their conversion to a bioactive metabolite. Up to 10% of drugs approved worldwide can be classified as prodrugs that are transformed by eukaryotic enzymes, such as esterases and phosphatases, or by non-enzymatic reactions driven by biochemical properties of the intracellular environment^20^. Using host cells to transform approved drugs into derivatives with antibacterial activity may render the drug inactive against its original human target, as is the case for LPZ and LPZS (Fig. 5)^9^. A substantial number of prodrugs can also be expected in small-molecule libraries containing compounds of unknown function. These molecules provide an untapped pool for antimicrobial drug discovery. Our strategy of host-mediated prodrug activation is generally applicable to other drug-resistant bacterial pathogens, thereby enabling inhibitors with novel targets to be found in compound libraries that have been interrogated previously in standard phenotypic screens.

## Methods

### Drugs used in this study

LPZ, omeprazole and pantoprazole were purchased from Sigma-Aldrich, LPZS and other LPZ analogues were purchased from Santa Cruz biotechnology, Toronto Research Chemicals Inc. and Alfa Aeser. Q203 was kindly provided by Kevin Pethe of the Institut Pasteur Korea. Other IPA compounds were derived from a GlaxoSmithKline library kindly provided by Lluis Ballell^21^.

### Culture conditions of *Mtb* and eukaryotic cell lines

Mycobacterial strains were routinely grown in Middlebrook 7H9 broth (supplemented with 0.2% glycerol, 10% albumin dextrose catalase (ADC) and 0.05% Tween-80) or 7H10 agar plates (supplemented with 0.5% glycerol and 10% oleic ADC). MRC-5 human lung fibroblasts were provided by the Coriell Institute for Medical Research and grown in MEM supplemented with 10% heat-inactivated fetal bovine serum, 1% non-essential amino acids and 1 mM sodium pyruvate. RAW264.7 macrophages derived from the EPFL strain collection were grown in RPMI medium supplemented with 10% fetal bovine serum. Both cell lines were grown at 37 °C with 5% CO_2_.

### High-throughput drug screen and intracellular assays

Compounds of the Prestwick chemical library were preplated into 384-well microplates (Corning) at a concentration of 100 μM in 5 μl of 5% dimethyl sulfoxide. MRC-5 cells grown to late-log phase were harvested and seeded at 4,000 cells per well in a volume of 35 μl using an automated microplate dispenser (multidrop combi, Thermo Scientific). Cells were allowed to adhere for 3 h and then were infected with washed logarithmic-phase *Mtb* Erdman cells at an MOI of 10 in 10 μl of MEM medium. Plates were sealed and incubated at 37 °C in 5% CO_2_. After 72 h, the plates were left at room temperature for 1 h and 5 μl of Prestoblue cell viability reagent (Life Technologies) were added. After 1 h at room temperature, fluorescence was measured in a Tecan Infinite M200 plate reader (excitation 570 nm and emission 590 nm). *Mtb* in infected macrophages or fibroblasts was quantified using an *Mtb* Erdman strain expressing EGFP and a Tecan plate reader (excitation 480 nm and emission 510 nm).

For fluorescence microscopy, RAW264.7 macrophages were seeded on round 9-mm coverslips in 24-well plates (10^5^ cells per well). To quantify intracellular *Mtb*, macrophages were infected at an MOI of 2 for 12 h. Cells were washed several times to remove unphagocytosed bacteria and fresh medium containing compounds or dimethyl sulfoxide was added. After incubation for 4 days, the cells were washed and fixed with 4% paraformaldehyde/PBS and stained with Dapi-Fluoromount-G (Southern Biotech). Images were acquired on a Zeiss LSM 700 using ZEN imaging software and Fiji processing software. Resazurin reduction microplate assays (REMAs) were performed in 7H9 broth using a starting OD_600_ of 0.0001, a 7-day (or 14 day) incubation period and a final volume of 10% resazurin (0.025% w/v). After incubation, fluorescence of the resazurin metabolite resorufin was measured (excitation at 570 nm and emission at 590 nm, gain 80) using a TECAN Infinite M200. For OD_600_-based MIC determination, bacterial OD_600_ was adjusted to 0.01 and bacteria were exposed to LPZS for 7 days followed by OD readings. For reading after 14 days of exposure, initial OD_600_ was adjusted to 0.0001. Drug testing against streptomycin-starved 18b was performed as described above for REMA assays using an SS18b culture maintained in 7H9 medium without streptomycin for 2 weeks and a final OD_600_ of 0.1 (ref. 15).

### ATP-depletion assay and ADP quantification

Log-phase *Mtb* cultures were exposed to drugs for 24 h and mixed with BacTiter-Glo reagent (v/v 4:1) (Promega) followed by incubation in the dark for 10 min. Luminescence (relative light units, RLUs) was read in a TECAN Infinite M200. For ADP detection and calculation of the ADP/ATP ratio, we exposed log-phase *Mtb* (OD_600_ of 0.01) to LPZS for 24 h in a white 96-well plate. BacTiter-Glo reagent (v/v 4:1) was added and luminescence was determined after 2 min (RLU_A_). Plates were incubated in the dark for 15 min and read again, which provided background ATP signals prior to ADP measurements (RLU_B_). Five microlitre of an ADP-converting enzyme (Sigma Alrich, MAK135E) were added, followed by a third measurement after 1 min of incubation (RLU_C_). The ADP/ATP ratio was calculated using the following formula: ADP/ATP=(RLU_C_−RLU_B_)/RLU_A_.

### Culture conditions and REMA assay of other microorganisms

Mycobacterium strains were grown in 7H9 broth (Difco) supplemented with Middlebrook ADC enrichment, 0.2% glycerol, 0.05% Tween-80. *Bacillus subtilis*, *Candida albicans*, *Corynebacterium glutamicum*, *Escherichia coli*, *Micrococcus luteus*, *Pseudomonas putida*, *Salmonella typhimurium* and *Staphylococcus aureus* were grown in Luria broth base (Sigma). *C. diphtheriae*, *Enterococcus faecalis*, *Listeria monocytogenes* and *P. aeruginosa* were grown in brain heart infusion broth (Difco). Twofold serial dilutions of each test compound were prepared in 96-well plates containing bacteria in a total volume of 100 μl and then incubated at 37 or 30 °C (as required) before addition of 10 μl of 0.025% resazurin. After incubation, fluorescence of the resazurin metabolite resorufin was determined as described above. Colony-forming unit (c.f.u.) counts and checkerboard assays for drug combination studies were performed using REMA assays and twofold serial dilutions of LPZS in combination with the drugs mentioned in Supplementary Table 5. The fractional inhibitory index (ΣFIC) was calculated as FIC (fractional inhibitory concentrations) of compound *X*+FIC of compound *Y* to evaluate interaction profiles. The FIC was calculated using the following formula: FIC (*X*+*Y*)=[MIC of compound *X* in combination with *Y*]/[MIC of *X* alone]. ΣFICs of ≤0.5 designate synergistic activity and values >0.5 additive activity^22^,^23^.

### Isolation and characterization of LPZS-resistant mutants

LPZS-resistant *Mtb* H37Rv mutants were isolated by plating 10^9^ c.f.u. on 7H10 agar containing 20 μM of LPZS. Genomic DNA was isolated as described recently^24^. Whole-genome sequencing was performed using Illumina technology with sequencing libraries prepared using the NEBnext Ultra DNA kit (New England Biolabs). The resulting genomic DNA fragment library was loaded on Illumina MiSeq Reagent Kit V3 cartridges and sequenced. All Illumina reads were right-trimmed to a length of 150 nucleotides to remove low-quality areas. Each sample was downsampled to 1.5-M read pairs to even the genome coverage between samples. Analysis was done with MIRA (version 4.0rc4) using the *Mtb* H37Rv genome (NC_000962.3) as reference. The resulting SNP table was parsed in Excel to identify informative SNPs.

### Recombineering method for target confirmation

*Mtb* H37Rv carrying plasmid pJV53 was grown to mid-log phase in 7H9 broth containing 25 μg ml^−1^ kanamycin and exposed to 0.2% acetamide for 16 h. Competent cells were co-transformed with 100 ng of single-stranded oligos (lagging and leading strand) (5′-CTCACTGCCTGACGACCTGCTGTCGGGACTCGGTCCGCGCGCGGCACTCTCGTCGATCACGCTGGGTATGC-3′—for the L176P mutation) or (5′-GGCGGGCTCGCAGCCAGACTTCTACATGATGTGGGCCGAGGGTCTGGCCCGGATCTGGCCGCCGTGGGAG-3′—for the T313A mutation) and pYUB412 (50 ng) followed by plating on 7H10 agar plates containing 50 μg ml^−1^ hygromycin. SNPs in hygromycin-resistant clones were confirmed by PCR and sequencing using oligos 5′-TGCTGATCACCGGCGTGTAT-3′ and 5′-AAGATGATCCCCGGCAACAG-3′ for L176P or 5′-TTCAAGTCCGGCGCATTTTT-3′ and 5′-TAGACGAACGGCGGCAGAAT-3′ for T313A.

### *Ex vivo* pharmacokinetics and ESI–MS

For intracellular drug quantification, 20,000 MRC-5 cells were seeded in 96-well plates and exposed to 2 μM LPZ. At given time points, cells were extensively washed with PBS and lysed with 0.1% Triton X-100 in PBS and acetonitrile (1:1 ratio). After spinning at 15,000*g* at 4 °C, samples were shock-frozen in liquid nitrogen and stored at −80 °C for MS. Quantification in 7H9 broth was performed with 500 nM of LPZ, acetonitrile was added in a 1:1 ratio at given time points followed by spinning and shock freezing. The UPLC separation was done on an Agilent 1290 Infinity LC system including the 1290 Infinity LC system binary pump with an integrated degasser, the high-performance autosampler and a thermostatted column compartment. Samples (2 μl) were injected into a Zorbax Extend-C18 (2.1 × 50 mm, 1.8 μm) analytical column (Agilent Technologies) operated at 40 °C, using H_2_O–HCOOH 0.1% and CH_3_CN–HCOOH 0.1% as mobile phases A and B, respectively, (2–100% B in 5 min) at a flow rate of 0.4 ml min^−1^. All samples were injected in duplicate. The UPLC system was interfaced with a 6530 Accurate-Mass Q-TOF LC/MS system (Agilent Technologies). ESI–MS data were acquired in the positive ionization mode, in the mass range *m/z* 100–1,000 (2 spectra s^−1^). Experimental parameters were set as follows: fragmentor: 190 V, Vcap: 3,500 V, gas temperature: 300 °C and sheath gas temperature: 350 °C. External calibration was carried out with a solution of ESI_L (Agilent). Data were processed using MassHunter quantitative analysis compliance software. Extracted ion chromatogram of ions at *m/z* 354.088 and 370.083 were integrated and quantified using external calibration of both LPZ and LPZS spiked in the same matrix.

### Mouse studies

For pharmacokinetic studies, female BALB/c mice (18–20 g) were given 100 mg kg^−1^ or 300 mg kg^−1^ LPZS in 20% TPGS (D-α tocopheryl polyethylene glycol 1,000 succinate) by gavage. At given time points, blood from three mice was taken and, after spinning, serum was treated with acetonitrile (1:4 ratio). After a second spin, supernatants were frozen for LC/MS experiments. For *in vivo* efficacy studies, mice were treated following the acute infection protocol of Rullas *et al.*^25^ with some modifications. In brief, female BALB/c mice (18–20 g; 4 mice per group) were aerosol infected with *Mtb* H37Rv and treated the following day with 300 mg kg^−1^ LPZS b.i.d. by gavage for 9 days. The day after final treatment, mice were killed and serial dilutions of lung homogenates were plated on 7H10 agar containing 10 μg ml^−1^ cycloheximide and 25 μg ml^−1^ ampicillin. Experiments were approved by the Swiss Cantonal Veterinary Authority (authorization number 2218).

### Statistical analysis

Student's *t*-test was used throughout the paper to compare two groups.

### Protein structure prediction

QcrB (Rv2196) from *M. tuberculosis* was modelled using the software iTasser ( http://zhanglab.ccmb.med.umich.edu/I-TASSER/) and the cytochrome *bc*_1_ structure from *R. sphaeroides* as template (PDB code 1QJY).

## Additional information

**Accession codes:** Genome sequencing data were deposited at the SRA database (NCBI) under the Study accession SRP049754.

**How to cite this article:** Rybniker, J. *et al.* Lansoprazole is an antituberculous prodrug targeting cytochrome *bc*_1_. *Nat. Commun.* 6:7659 doi: 10.1038/ncomms8659 (2015).

## Supplementary Material

Supplementary Information Supplementary Figures 1-7 and Supplementary Tables 1-5

## Figures and Tables

**Table 1:** Activity of LPZS (in μM) against selected microorganisms.

**Pathogen**	**MIC**_**90**_ **day-7 REMA**	**MIC**_**90**_ **day-7 OD**_**600**_	**MIC**_**90**_ **day-14 REMA**	**MIC**_**90**_ **day-14 OD**_**600**_
*Mycobacterium tuberculosis* H37Rv	1.13	1.02	1.71	1.34
*Mycobacterium tuberculosis* Erdman	1.21			
*Mycobacterium tuberculosis* HN878 (Beijing strain)	1.74			
*Mycobacterium abcessus* 2005-0524	>100			
*Mycobacterium avium*	>100			
*Mycobacterium bolletii* 1999-0888	>100			
*Mycobacterium marinum* M	100			
*Mycobacterium massiliense* 2005-0484	>100			
*Mycobacterium smegmatis* mc^2^155	>100			
*Mycobacterium vaccae* ATCC 15483	>100			
*Pseudomonas aeruginosa*	>100			
*Pseudomonas putida*	>100			
*Salmonella typhimurium*	>100			
*Staphylococcus aureus*	>100			
*Bacillus subtilis*	>100			
*Candida albicans*	>100			
*Corynebacterium diphtheriae*	>100			
*Corynebacterium glutamicum*	>100			
*Enterococcus faecalis*	>100			
*Escherichia coli*	>100			
*Listeria monocytogenes*	>100			
*Micrococcus luteus*	>100			

LPZS, lansoprazole sulfide; MIC, minimal inhibitory concentration; REMA, resazurin reduction microplate assay.

Activity of LPZS is highly selective for *Mtb*. The MIC of the *Mtb* H37Rv strain was determined by REMA assays and OD_600_ measurements after 7 and 14 days of LPZS exposure. Both methods gave similar results.

**Table 2:** Activity of LPZS against drug-resistant clinical isolates of *Mtb*.

**Clinical isolate ID**	**Resistance**	**MIC**_**90**_ **(μM)**
*M. tuberculosis* 59744	INH, RIF	0.78
*M. tuberculosis* MB3649	INH	1.37
*M. tuberculosis* MI1020	INH, STR	0.94
*M. tuberculosis* 43061	INH	0.49
*M. tuberculosis* 45776	INH	0.52
*M. tuberculosis* 49975	INH	1.06

INH, isoniazid; LPZS, lansoprazole sulfide; RIF, rifampicin; STR, streptomycin.
