# Supplementary material for: Lansoprazole is an antituberculous prodrug targeting cytochrome bc1
Source: Nat Commun. 2015 Jul 9;6:7659. doi: 10.1038/ncomms8659 (PMC4510652; doi:10.1038/ncomms8659)
Supplement: Supplementary Information — Supplementary Figures 1-7 and Supplementary Tables 1-5 [file ncomms8659-s1.pdf]

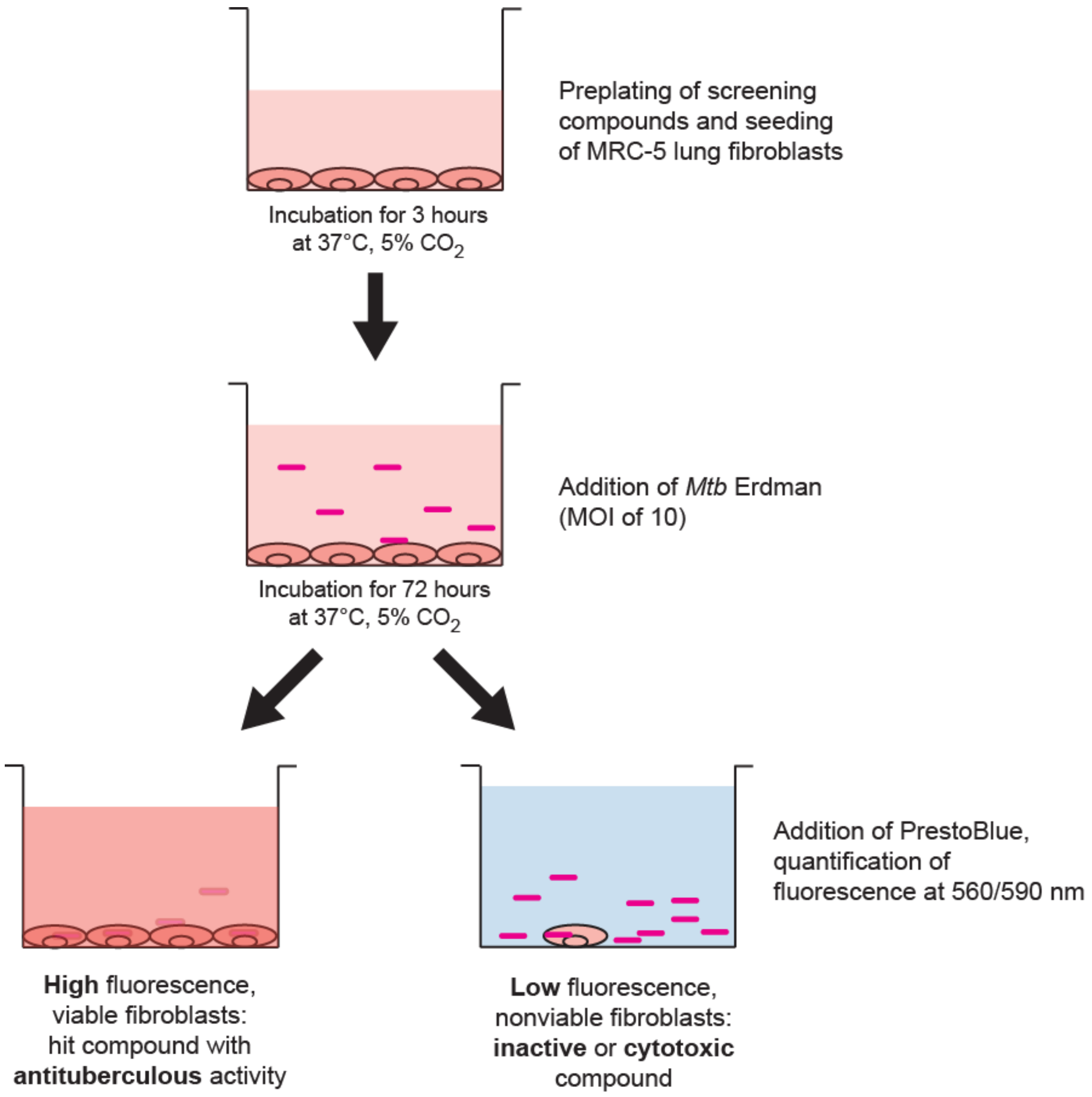


**Supplementary Figure 1. Flow diagram of the host cell-based HTS used in this study.** Due to the rapid turnover of PrestoBlue by eukaryotic cells, background fluorescence from bacteria is negligible. A full description of the assay can be found in the methods section. MOI: multiplicity of infection.

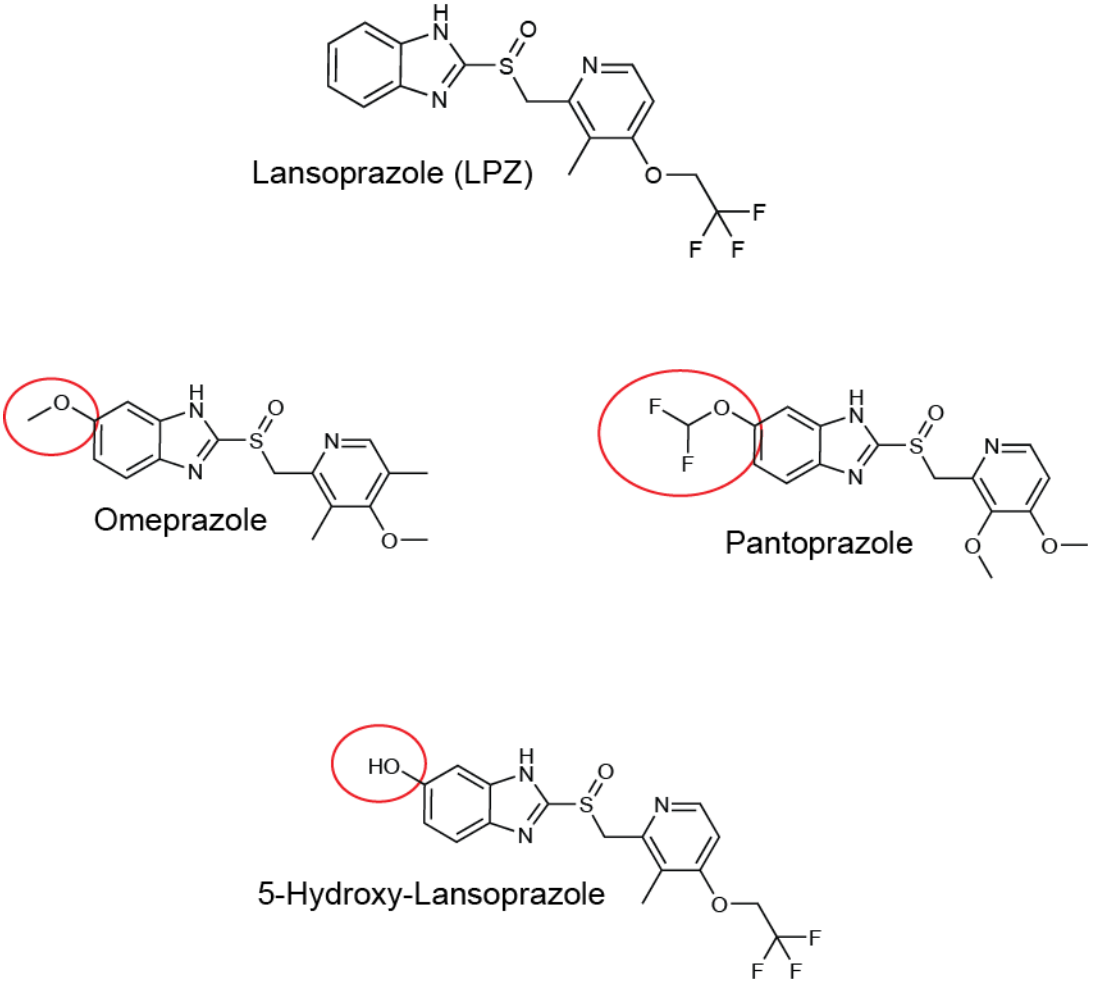


**Supplementary Figure 2**. **Structures of different proton pump inhibitors and the LPZ metabolite 5-Hydroxy-Lansoprazole**. Molecules carrying substitutions on the benzimidazole ring (red circle) are not active against *Mtb*.

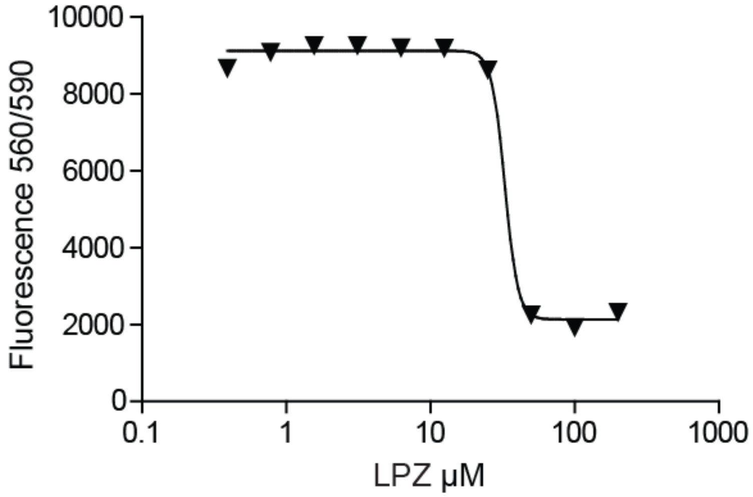


**Supplementary Figure 3. Dose-response curve of LPZ against *Mtb* H37Rv in 7H9 broth.** Representative example of three individual experiments (mean ± SD of duplicates).

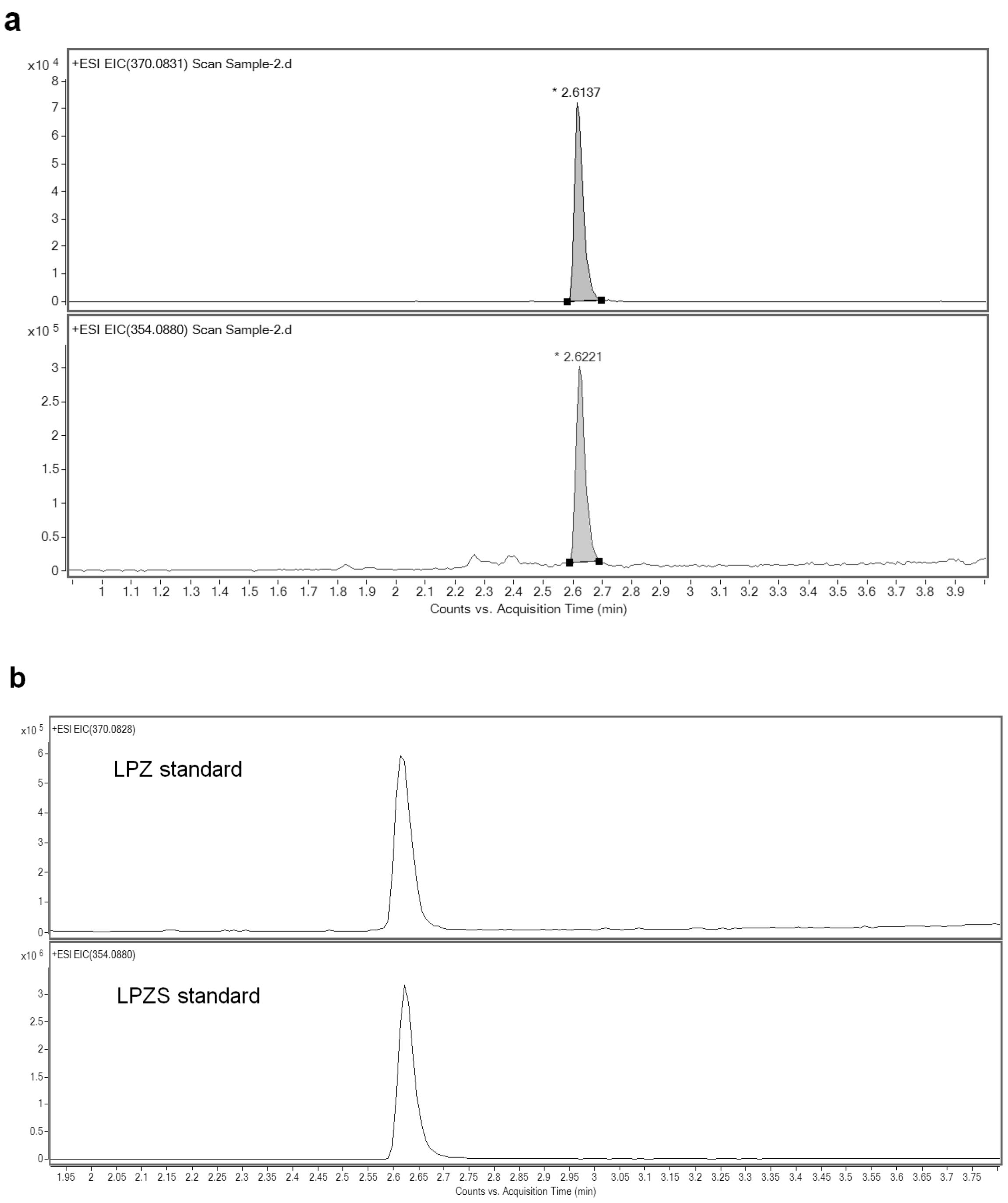


**Supplementary Figure 4. Extracted Ion Chromatograms (EIC). (a)** EIC of *m/z* 354.088 and 370.083 derived from a cell lysate sample of MRC-5 cells treated with LPZ. **(b)** EIC of *m/z* 354.088 and 370.083 corresponding respectively to the LPZ standard and the LPZS standard.

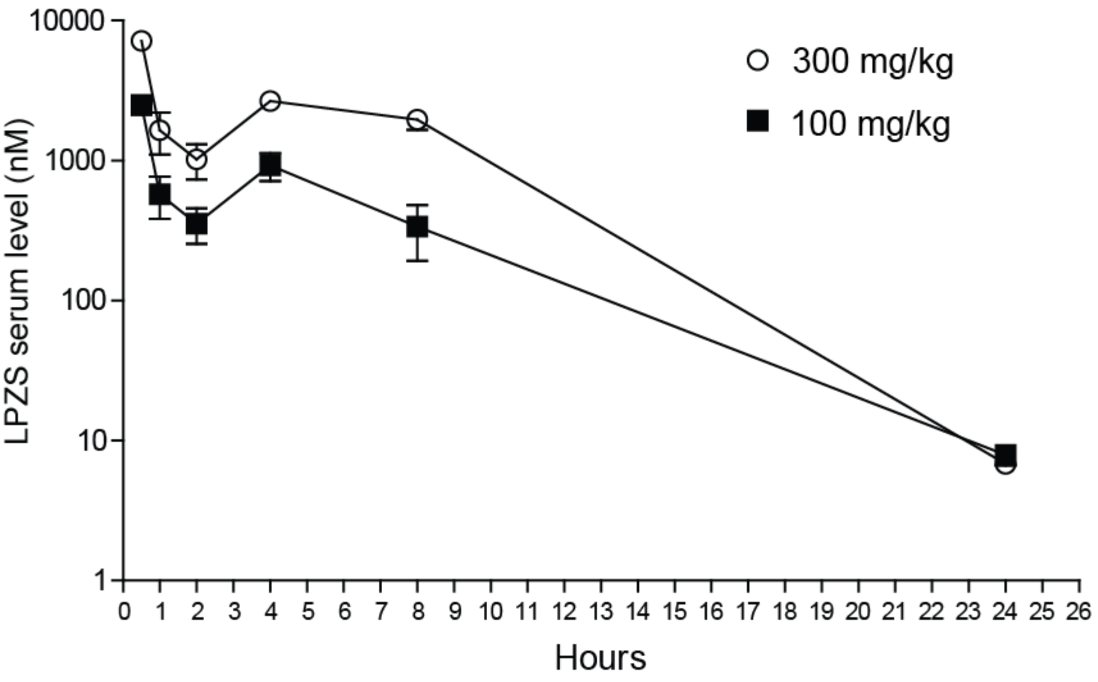


**Supplementary Figure 5. *In vivo* pharmacokinetics of LPZS.** BALB/c mice (three per group) were given 100 mg/kg or 300 mg/kg of LPZS by oral gavage and serum levels were determined after 0.5, 1, 2, 4, 8 and 24 hours (mean ± SD). LPZS shows non-linear pharmacokinetics with a second peak after approximately 4 hours of administration.

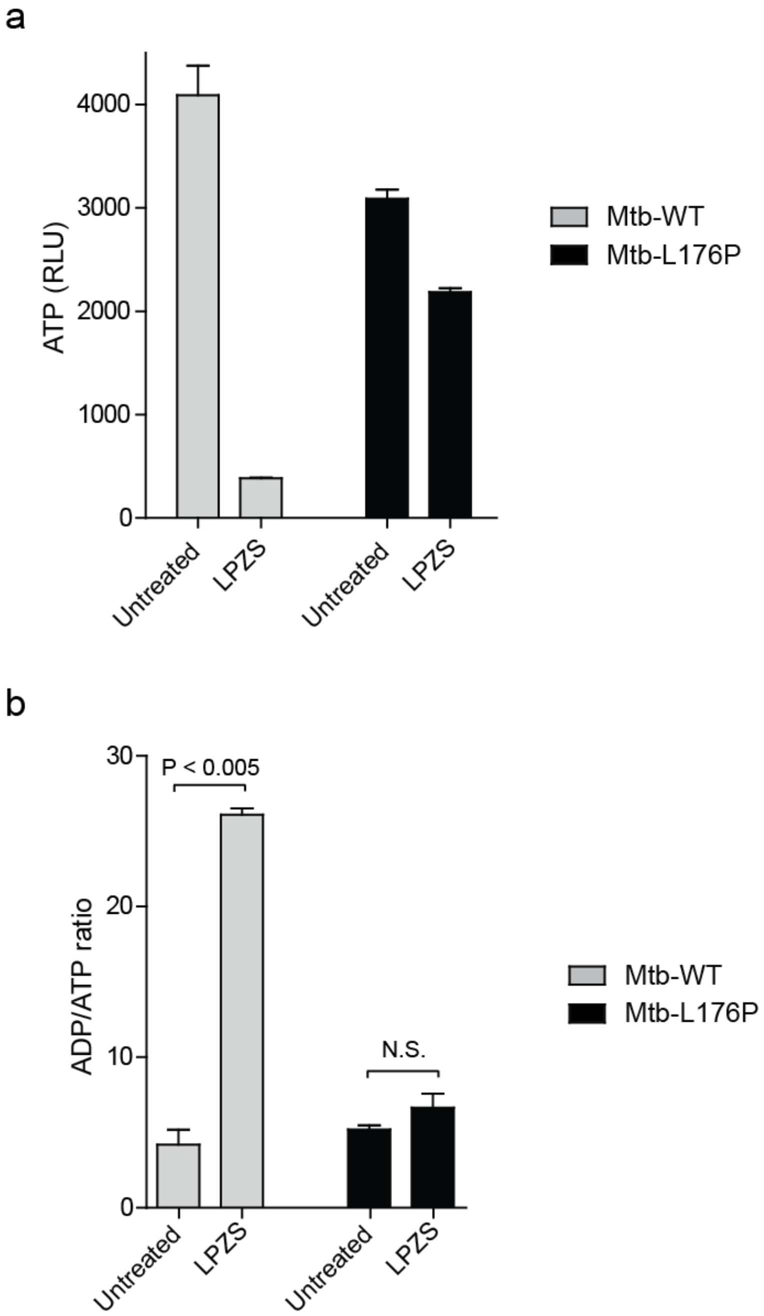


**Supplementary Figure 6. ATP depletion and ADP/ATP ratios in wild-type and L176P mutant strain. (a)** ATP levels are only slightly affected in the LPZS treated L176P mutant strain. **(b)** The ADP/ATP ratio of LPZS treated wild-type bacteria shifts several fold in comparison to untreated bacteria. A similar effect was not observed for the L176P mutant. LPZS concentration was 5 µM (mean ± SD of three individual experiments; unpaired Student's *t* test was used to compare groups).

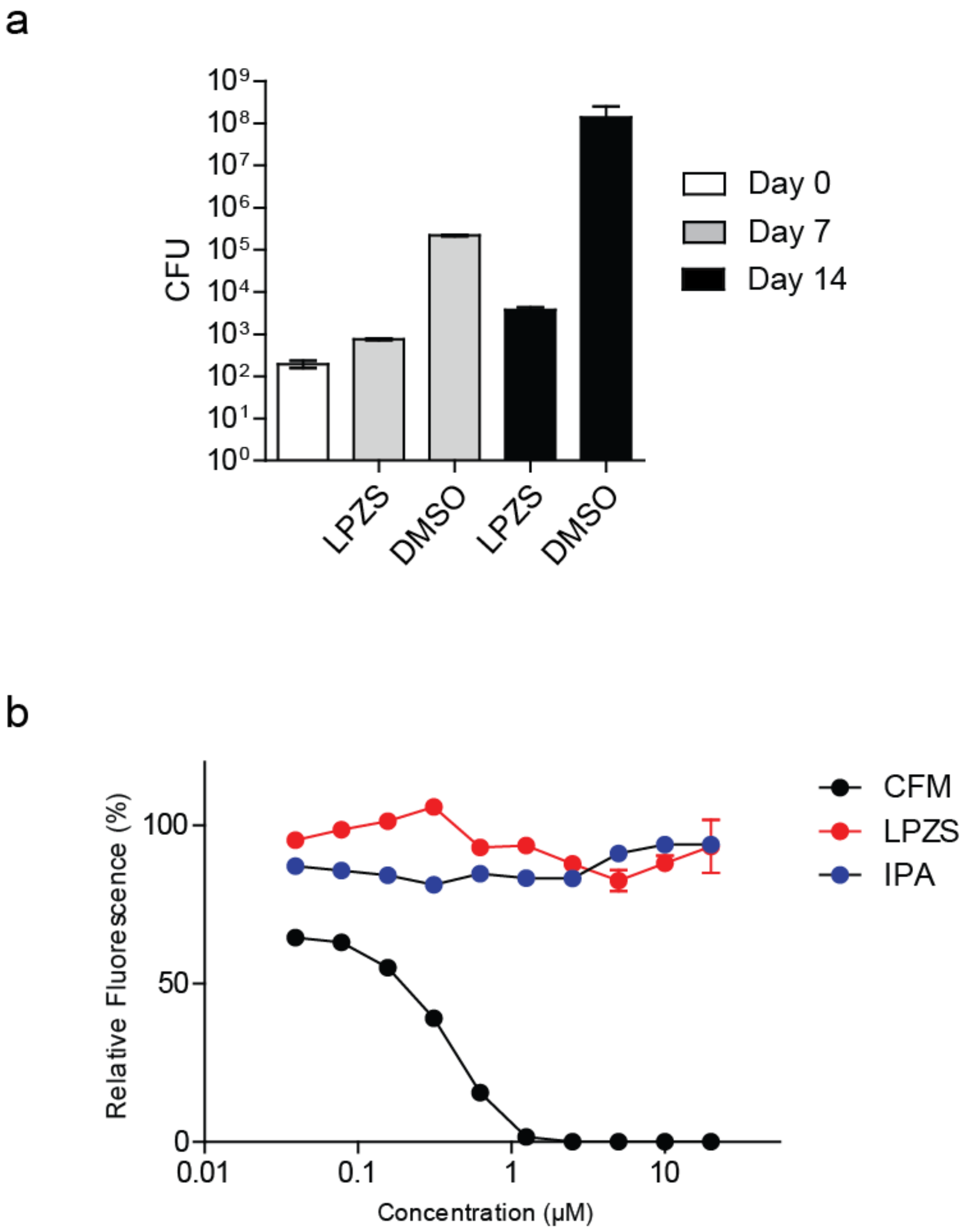


**Supplementary Figure 7. CFU counts for LPZS treated bacteria and *Mtb*-18b assays. (a)** *Mtb* H37Rv was exposed to LPZS and serial dilutions were plated for CFU counts after 7 and 14 days. The data show that LPZS is a bacteriostatic compound. **(b)** LPZS and an imidazopyridine amide (IPA) compound were tested in the non-replicating, SS18b model using clofazimine (CFM) as a positive control (mean ± SD of two individual experiments).

**Supplementary Table 1. Hit list of the Prestwick chemical library screen.**

The library contained 1,280 FDA-approved drugs. Shown are hit-compounds with known antimicrobial activity and lansoprazole. The score value is relative to the control rifampicin (value of 1). Score SD represents the standard deviation from measurements of duplicate assay plates.

| Catalog-ID | Name | Score | Score SD |
|---|---|---|---|
| Prestw-28 | Ethambutol dihydrochloride | 1,225814 | 0,033595 |
| Prestw-1109 | Rifabutin | 1,189656 | 0,044552 |
| Prestw-1429 | Linezolid | 1,188858 | 0,030995 |
| Prestw-1478 | Rifaximin | 1,035642 | 0,001289 |
| Prestw-1157 | Rifapentine | 1,019149 | 0,089935 |
| Prestw-127 | Isoconazole | 0,932132 | 0,10216 |
| Prestw-1321 | Prothionamide | 0,923799 | 0,051977 |
| Prestw-161 | Isoniazid | 0,918147 | 0,019877 |
| Prestw-525 | Rifampicin | 0,911659 | 0,011737 |
| Prestw-526 | Ethionamide | 0,878031 | 0,038328 |
| Prestw-67 | Miconazole | 0,708215 | 0,008418 |
| Prestw-1446 | Moxifloxacin | 0,700677 | 0,02008 |
| Prestw-787 | Merbromin | 0,690025 | 0,082574 |
| Prestw-390 | Fusidic acid sodium salt | 0,659256 | 0,103686 |
| Prestw-1072 | Lansoprazole | 0,633754 | 0,02461 |
| Prestw-1378 | Clarithromycin | 0,633549 | 0,107855 |
| Prestw-1184 | Tioconazole | 0,602143 | 0,131729 |
| Prestw-1265 | Gatifloxacin | 0,59116 | 0,04248 |
| Prestw-854 | Roxithromycin | 0,585573 | 0,002029 |
| Prestw-143 | Chlorhexidine | 0,563608 | 0,112897 |
| Prestw-456 | Meclocycline sulfosalicylate | 0,558025 | 0,033866 |
| Prestw-753 | Demeclocycline hydrochloride | 0,540454 | 0,028363 |
| Prestw-1202 | 4-aminosalicylic acid | 0,504281 | 0,021594 |
| Prestw-1469 | Mercaptopurine | 0,49779 | 0,020288 |
| Prestw-1353 | Tylosin | 0,484304 | 0,068005 |
| Prestw-964 | Methacycline hydrochloride | 0,479672 | 0,012353 |
| Prestw-1399 | Doxycycline hydrochloride | 0,467443 | 0,043805 |
| Prestw-1234 | Azithromycin | 0,457259 | 0,002645 |
| Prestw-304 | Econazole nitrate | 0,435142 | 0,01197 |
| Prestw-1340 | Secnidazole | 0,410823 | 0,08413 |
| Prestw-154 | Josamycin | 0,389697 | 0,006481 |
| Prestw-867 | Paromomycin sulfate | 0,379752 | 0,006313 |
| Prestw-376 | Clofazimine | 0,372465 | 0,012093 |
| Prestw-159 | Dihydrostreptomycin sulfate | 0,371586 | 0,033046 |
| Prestw-222 | Antimycin A | 0,369432 | 0,009911 |
| Prestw-1241 | Bifonazole | 0,340554 | 0,020859 |
| Prestw-389 | Ketoconazole | 0,315344 | 0,033782 |

| | | | |
|---|---|---|---|
| Prestw-151 | Erythromycin | 0,304405 | 0,005892 |
| Prestw-1118 | Cefepime hydrochloride | 0,304193 | 0,078126 |
| Prestw-31 | Chloramphenicol | 0,277552 | 0,016772 |
| Prestw-1113 | Viomycin sulfate | 0,267181 | 0,051968 |
| Prestw-1005 | Apramycin | 0,264068 | 0,0378 |
| Prestw-557 | Dirithromycin | 0,263181 | 0,00337 |

**Supplementary Table 2. Ratios of LPZ and LPZS**

(a) Intracellular ratio of LPZ (*m/z* 370.0834 g/mol) and its metabolite (*m/z* 354.0884 g/mol) determined by electrospray ionisation mass spectrometry (ESI-Q-TOF-MS) over a 48 hour period in MRC-5 cells. (b) LPZ/LPZS ratio determined by ESI-Q-TOF-MS over a 48 hour period in 7H9 broth.

**A:**

| | Replicate 1 | | Replicate 2 | | Replicate 3 | |
|---|---|---|---|---|---|---|
| | LPZ | LPZS | LPZ | LPZS | LPZ | LPZS |
| **1 hour** | 1,000 | 0,0885 | 1,000 | 0,0767 | 1,000 | 0,091 |
| **5 hours** | 0,360 | 0,3030 | 0,281 | 0,4210 | 0,311 | 0,313 |
| **24 hours** | 0,136 | 0,8240 | 0,120 | 0,9100 | 0,210 | 0,681 |
| **48 hours** | 0,021 | 0,9001 | 0,011 | 0,9300 | 0,017 | 0,811 |

**B:**

| | Replicate 1 | | Replicate 2 | | Replicate 3 | |
|---|---|---|---|---|---|---|
| | LPZ | LPZS | LPZ | LPZS | LPZ | LPZS |
| **0 hours** | 1,000 | 0,000125 | 1,000 | 0,000090 | 1,000 | 0,000190 |
| **1 hour** | 0,904 | 0,004800 | 0,871 | 0,009100 | 0,870 | 0,006100 |
| **5 hours** | 0,580 | 0,017000 | 0,500 | 0,051000 | 0,465 | 0,021000 |
| **24 hours** | 0,089 | 0,039000 | 0,081 | 0,061000 | 0,051 | 0,034000 |
| **48 hours** | 0,020 | 0,045000 | 0,028 | 0,054000 | 0,012 | 0,061000 |

**Supplementary Table 3. Cytotoxicity of LPZS on human cell lines.**

Cytotoxicity of LPZS on THP1 macrophages, human liver hepatoma cells (HuH7-D12), human lung epithelial carcinoma cells (A549) and human embryonic liver cells (HepG2).

| | **THP-1** | | **HuH7-D12** | | **A549** | | **HepG2** | |
|---|---|---|---|---|---|---|---|---|
| | TD99 | TD50 | TD99 | TD50 | TD99 | TD50 | TD99 | TD50 |
| **LPZS (µM)** | >100 | 75 | >100 | >100 | >100 | 37.5 | 100 | 25 |

**Supplementary Table 4. Drug combination studies.** LPZS acts additively with first- and second-line anti-TB drugs. ΣFIC: fractional inhibitory concentration indices

| **Compound** | **ΣFIC** | **Outcome** |
|---|---|---|
| Rifampicin | 1 | Additive |
| Isoniazid | 1 | Additive |
| Moxifloxacin | 1 | Additive |
| Bedaquiline | 1 | Additive |
| BTZ043 | 0.75 | Additive |

**Supplementary Table 5. The L176P mutation has no effect on imidazopyridine amide (IPA) activity.** This stands in contrast to the T313A mutation which confers high level resistance towards IPA-compounds.

| | **MIC (µM)** | | |
|---|---|---|---|
| **IPA compound** | **Wild-type** | **L176P** | **T313A** |
| **GSK2111534A** | 0.4 | 0.4 | > 25 |
| **GSK1829820A** | 0.2 | 0.2 | > 25 |
| **GSK1829736A** | 0.8 | 0.8 | > 25 |
| **Q203** | 0.006 | 0.012 | > 0.1 |
